# Multiorgan Failure Resembling Grade 5 (Fatal) Cytokine Release Syndrome in Patient with Multiple Myeloma Following Carfilzomib Infusion: A Case Report

**DOI:** 10.3390/jcm14134723

**Published:** 2025-07-03

**Authors:** Strahinja Gligorevic, Nebojsa Brezic, Joshua Jagodzinski, Andjela Radulovic, Aleksandar Peranovic, Igor Dumic

**Affiliations:** 1 Internal Medicine Residency Program, North Central Bronx Hospital, Bronx, NY 10467, USA; strahinja.gligorevic99@gmail.com; 2 Internal Medicine Residency Program (Preliminary Track), Lincoln Medical and Mental Health Center, Bronx, NY 10467, USA; nebojsabrezic@gmail.com; 3 Hospital Medicine Fellowship, Mayo Clinic Health System, Eau Claire, WI 54703, USA; jagodzinski.joshua@mayo.edu; 4 School of Medicine, University of Novi Sad, Novi Sad 400111, Serbia; 300952@mf.uns.ac.rs (A.R.); 301054@mf.uns.ac.rs (A.P.); 5 Department of Hospital Medicine, Mayo Clinic Health System, Eau Claire, WI 54703, USA

**Keywords:** carfilzomib, cytokine release syndrome, multiorgan failure, shock, multiple myeloma

## Abstract

**Background:** Cytokine release syndrome (CRS) is a life-threatening systemic inflammatory condition marked by excessive cytokine production, leading to multi-organ dysfunction. It is commonly associated with T-cell-engaging therapies such as chimeric antigen receptor (CAR) T cells, T-cell receptor bispecific molecules, and monoclonal antibodies. Carfilzomib, a proteasome inhibitor, is known to cause a range of adverse effects, primarily hematologic and cardiovascular. However, multiorgan failure grade 5 (fatal), resembling CRS has not been previously reported in association with Carfilzomib. **Case Report**: A 74-year-old male with relapsed multiple myeloma developed grade 5 multiorgan failure 60 min after the third dose of Carfilzomib, resulting in death within 24 h of symptom onset. The patient tolerated the first doses of Carfilzomib well with only fever and headache developing post infusion. Before the second dose, the patient developed worsening pancytopenia, prompting the discontinuation of Lenalidomide. After the second Carfilzomib infusion, he experienced fever and transient encephalopathy, which resolved with acetaminophen, corticosteroids, and supportive care. However, following the third dose, he rapidly deteriorated—developing fever, tachycardia, hypotension, hypoxia, and encephalopathy. Despite aggressive management with intravenous fluids, broad-spectrum antibiotics, corticosteroids, and tocilizumab, the patient progressed to refractory shock and multi-organ failure, culminating in death within 24 h. A comprehensive infectious workup was negative, ruling out sepsis and suggesting possible Carfilzomib-induced CRS. **Conclusion:** Grade 5 multiorgan failure with signs and symptoms similar with CRS following Carfilzomib administration is a rare but potentially fatal adverse drug reaction. Further research is needed to better define the risk factors and optimal management strategies for Carfilzomib-induced multiorgan failure and possible CRS.

## 1. Introduction

Cytokine release syndrome (CRS) is a complex and not yet fully understood systemic inflammatory response that can be life-threatening. It is triggered by a rapid and massive release of cytokines, primarily from activated macrophages and other immune cells. CRS typically presents with non-specific symptoms such as fever, fatigue, myalgia, and rash, and may progress to varying degrees of pulmonary, renal, cardiac, and neurological involvement. Severe, life-threatening cases of CRS are characterized by high fever, hypotension, circulatory shock, and multi-organ failure, including acute respiratory distress syndrome (ARDS) and disseminated intravascular coagulation (DIC), among other complications [1,2].

CRS is commonly observed in association with CAR-T cell therapy [3], as well as during treatment with tebentafusp for metastatic uveal melanoma [4]. It can also occur with various immunotherapies, including nivolumab, ipilimumab, and durvalumab [5]. In some cases, CRS may develop during viral infections such as influenza and has been reported as a complication of SARS-CoV-2 infection, among others. Diagnosis of CRS is clinical and relies on recognition within the appropriate epidemiological context. It is supported by comprehensive laboratory evaluation and the exclusion of other potential causes, primarily infectious and inflammatory conditions.

Carfilzomib, a selective proteasome inhibitor used in the treatment of relapsed or refractory multiple myeloma (MM) following progression on one or more lines of therapy, exerts its anti-cancer effect by irreversibly binding to the 20S proteasome, thereby halting cellular proliferation and inducing apoptosis in malignant cells. However, its clinical utility is tempered by a broad spectrum of adverse effects. Hematologic toxicities, including anemia, thrombocytopenia, and lymphopenia, are among the most common [6]. Non-hematologic side effects such as fatigue, nausea, diarrhea, dyspnea, and fever are frequently reported, but more serious complications pose significant challenges. These include cardiovascular events like heart failure [6], coronary vasospasm [7], pulmonary hypertension [8,9], acute renal failure [5], acute lung injury [10], and rare yet life-threatening conditions such as thrombotic microangiopathy (TMA), which may necessitate treatment discontinuation and initiation of therapies like eculizumab [11,12,13].

## 2. Case Report

The patient is a 74-year-old male with a history of monoclonal gammopathy of undetermined significance (MGUS), diagnosed 20 years ago. Approximately 18 months prior to his most recent admission, the patient presented with worsening pancytopenia and increase in serum creatinine from a baseline value of 0.9 mg/dL to 1.7 mg/dL. A workup for MGUS progression revealed findings suggestive of multiple myeloma. Notable laboratory abnormalities included an increase in the lambda free light chain level to 180 mg/L (from 22.3 mg/L), an elevated immunoglobulin G (IgG) level of 3837 mg/dL (from 1332 mg/dL), and an M spike of 3.5 g/dL (from 0.5 g/dL). Given the significant anemia, gastrointestinal bleeding was ruled out through normal esophagogastroduodenoscopy (EGD) and colonoscopy. Bone marrow flow cytometry was negative for B-cell malignancy and increased blasts. However, plasma cell flow cytometry demonstrated monotypic lambda plasma cells, with an S-phase of 4%.

The patient was initiated on a combination treatment regimen consisting of Daratumumab, Bortezomib, and Dexamethasone in a 28-day cycle. He successfully completed 15 cycles, with a favorable response. However, 2 months after completing the initial chemotherapy, the patient experienced a relapse, as evidenced by an increase in lambda free light chains to 78.8 mg/L (from a nadir of 1.11 mg/L) and a rising M spike to 1.0 g/dL (from a nadir of undetectable levels). Consequently, he was switched to a new treatment regimen consisting of Carfilzomib, Lenalidomide, and Dexamethasone. He tolerated the first dose well, apart from a fever of 38.1 °C, which was successfully managed with acetaminophen. Prior to the second dose, the patient developed worsening pancytopenia, leading to a temporary hold on Lenalidomide. He proceeded to receive Carfilzomib and Dexamethasone alone.

The second dose was complicated by fever and mild hypotension, transient confusion, which were managed with 1 L of intravenous (IV) normal saline, IV acetaminophen, and an additional dose of IV methylprednisolone, resulting in improvement in mental status. No evidence of hypoxia, or arrhythmia was noted. The patient subsequently presented for the third dose of Carfilzomib, which was administered at a dose of 110 mg IV, accompanied by 125 mg of IV methylprednisolone and 25 mg of IV diphenhydramine as premedication. Full details regarding the dosing schedule, symptoms, laboratory values, treatment and outcome of treatment are presented in Table 1.

Approximately 60 min after completion of the third infusion, the patient developed a fever of 39 °C, tachycardia (heart rate 110 beats per minute), tachypnea (30 breaths per minute), hypoxia (85% on ambient air), and worsening encephalopathy. He was promptly transferred from the oncology infusion clinic to the emergency department and was eventually admitted to a critical care unit given hemodynamic instability and need for vasopressors and mechanical ventilation.

Aggressive resuscitation was initiated with intravenous fluids, and broad-spectrum antimicrobial therapy was initiated, including ceftriaxone 2 g every 12 h, vancomycin 15 mg/kg every 12 h, doxycycline 100 mg IV twice daily, and acyclovir 10 mg/kg every 8 h. He also received a single dose of micafungin 100 mg empirically for sepsis. Despite these interventions, the patient’s condition deteriorated, and he developed hypotension unresponsive to IV fluid resuscitation, necessitating the initiation of norepinephrine infusion. Stress doses of corticosteroids were administered (hydrocortisone 100 mg IV every 6 h), and the patient was started on tocilizumab, given the clinical suspicion of cytokine release syndrome (CRS). Head CT was obtained emergently, and it was negative for any acute intracranial process. Due to hemodynamic instability, obtaining brain MRI was not feasible

Despite the addition of vasopressin and epinephrine infusions, the patient’s hypotension continued to worsen, and his respiratory status further deteriorated, leading to acute hypoxemic respiratory failure with bilateral infiltrates on chest X-ray, suggestive of acute respiratory distress syndrome (ARDS). At that point trachea was intubated. A bedside echocardiogram revealed a preserved ejection fraction, with no wall motion abnormalities or pericardial effusion. Comprehensive infectious work up including blood cultures, bronchoalveolar lavage cultures, cerebrospinal fluid analysis and inflammatory/autoimmune work up was initiated. Unfortunately, after 24 h following his third dose of Carfilzomib patient died from grade 5 CRS. Comprehensive work up to exclude other possible conditions is illustrated in Table 2. The patient’s family declined autopsy.

## 3. Discussion

Cytokine release syndrome (CRS) is an acute systemic inflammatory condition characterized by fever and varying degrees of multi-organ dysfunction. It is associated with CAR-T cell therapy but has also been observed with other immunotherapies and stem cell transplantation. CRS is among the most frequently reported side effects of tebentafusp—a T-cell receptor-bispecific molecule targeting glycoprotein 100 and CD3 [2,4,14]. Although not classified as an immunotherapy, Carfilzomib has been reported to induce infusion-related cytokine release. Clinical trials have described mild “flu-like” symptoms, such as fever and chills, however, severe CRS-like reactions are exceedingly rare [5]. Fatal CRS directly attributed to Carfilzomib has not been reported to date.

The American Society for Transplantation and Cellular Therapy (ASTCT) Consensus Grading for CRS categorizes severity based on fever, hypotension, and hypoxia. Grade 1 CRS is defined as fever (≥38 °C) without hypotension or hypoxia, while Grade 2 involves fever with hypotension responsive to fluids or hypoxia requiring low-flow oxygen. Grade 3 is characterized by hypotension requiring a single vasopressor or hypoxia requiring high-flow oxygen, whereas Grade 4 represents life-threatening symptoms necessitating multiple vasopressors or mechanical ventilation. Grade 5 CRS is fatal [15]. Severe cases (Grade ≥ 3) occur in approximately 5% of myeloma trials [3] and up to 20–30% in early acute lymphoblastic leukemia (ALL) studies [16].

CAR T-related CRS is frequently severe (above grade 2) with high fever, tachycardia, hypotension, and hypoxia, sometimes progressing to multi-organ failure [16]. In contrast, monoclonal antibody therapies generally cause milder infusion reactions; for instance, daratumumab (anti-CD38) induces CRS-like symptoms in approximately 46% of patients, mostly Grade 1–2, with severe reactions occurring in about 3% [17]. Tebentafusp causes CRS in up to 86% of patients but these are usually mild cases (Grade 1 and 2) and occur within 24 h post infusion [4]. Blinatumomab (anti-CD19/CD3) can also trigger CRS, though prophylactic steroids have mitigated its severity, reducing the incidence of Grade ≥ 3 CRS to 2% in adult ALL trials [16].

Although daratumumab is well known for causing infusion-related reactions [18], our patient had received the last dose more than three months prior to the onset of CRS, making it an unlikely contributing factor. Our patient developed grade 5 multiorgan failure resembling CRS and succumbed to it, despite receiving IV steroids for prophylaxis. Compared to CAR T or T-cell engagers, the risk of CRS with Carfilzomib is significantly lower, making the fulminant reaction in this case an outlier that underscores the distinct differences in expected severity and poses challenges to timely and accurate diagnosis.

Diagnosing CRS in the context of Carfilzomib therapy is challenging, as it is an unexpected toxicity that mimics other acute conditions. Unlike in immunotherapy settings, where CRS is anticipated and systematically graded [15], clinicians do not routinely suspect it with Carfilzomib. The clinical presentation often resembles sepsis, with fever, tachycardia, hypotension, and organ dysfunction, fulfilling systemic inflammatory response criteria [19]. Ruling out infectious causes is mandatory while administering broad spectrum antimicrobial therapy. CRS and septic shock share overlapping hemodynamic and inflammatory profiles, both capable of inducing vasodilatory shock, capillary leak, and multi-organ failure [19]. CRS cytokine profiles also overlap with macrophage activation syndrome/hemophagocytic lymphohistiocytosis (MAS/HLH) [20].

Patients like ours, who are receiving cancer directed therapy are at increased risk for developing infection given underlying immunosuppression and in such patients, sepsis must be ruled out and empiric antimicrobial therapy should be initiated as soon as possible. CRS lacks a definitive test, and inflammatory markers such as IL-6, CRP, and ferritin are nonspecific, also rising in infections [21,22,23]. Diagnosis relies on exclusion and pattern recognition: key indicators include timing (CRS typically follows infusion and up to 24 h after that—as seen in our case developing about 60 min post-Carfilzomib infusion), whereas sepsis occurs independently [19]. The absence of an infectious source (negative infectious work up and lack of response to antibiotics) further supported a non-infectious inflammatory process. Given the limited awareness of possible CRS following Carfilzomib infusion the recognition and treatment might be delayed. Table 3 lists the most important differential diagnosis consideration in a febrile patient with pancytopenia and evidence of SIRS.

In addition to profound shock, our patient developed two noteworthy complications that warrant further elaboration: acute respiratory distress syndrome (ARDS) and encephalopathy. ARDS has been previously reported in association with Carfilzomib therapy and may result from an interplay of pulmonary endothelial injury, immune activation, and systemic inflammatory responses. A case described by Ghasoub et al. detailed a patient developing ARDS shortly after receiving Carfilzomib, highlighting this rare but serious pulmonary complication and emphasizing the need for heightened clinical vigilance during Carfilzomib treatment [10]. The encephalopathy observed in our patient was most likely related to cytokine release syndrome (CRS), as it developed in close temporal proximity to systemic inflammatory manifestations. Sepsis itself is a well-known cause of encephalopathy, and differentiating between sepsis-associated encephalopathy and other etiologies can be clinically challenging [22]. As we ruled out sepsis by negative infectious work up and lack of response to broad spectrum antimicrobial therapy, we believe that encephalopathy might be part of CRS. In the setting of CAR T-cell therapy, a distinct neurotoxicity known as immune effector cell-associated neurotoxicity syndrome (ICANS) has been identified. ICANS is characterized by symptoms such as confusion, aphasia, and seizures—often occurring alongside cytokine release syndrome (CRS) and shares features with other forms of inflammatory encephalopathy. Although our patient did not receive CAR T-cell therapy, it’s important to consider that similar pathophysiological mechanisms may contribute to neurotoxicity associated with other immune-modulating agents, such as proteasome inhibitors. Recognizing both the similarities and differences in clinical presentation among these conditions is essential for accurate diagnosis and management. Carfilzomib-induced encephalopathy, microangiopathy, and endothelial dysfunction are believed to result from the drug’s inhibition of the proteasome, which disrupts protein homeostasis in endothelial cells. This disruption can lead to endothelial injury and promote microvascular thrombosis. The resulting endothelial dysfunction and microvascular compromise may manifest as encephalopathy.

Management of possible Carfilzomib-induced CRS follows general CRS principles but is based on limited clinical experience. Prevention of CRS with dexamethasone premedication (4–8 mg) is recommended [5], although severe multiorgan failure can still occur, as demonstrated in our case. Unlike CRS associated with CAR-T therapy, which follows standardized grading-based protocols with routine tocilizumab and steroids [16], or Blinatumomab-induced CRS, which is managed by stopping the infusion and administering dexamethasone [16], multiorgan failure potentially due to Carfilzomib CRS lacks established treatment guidelines, owing to rarity of this adverse event.

The prognosis of CRS varies by therapy and response to treatment. In expected settings such as CAR-T therapy, where CRS is managed promptly with tocilizumab and steroids, mortality is low (<5%), and most patients recover within a week [24]. Blinatumomab-induced CRS typically resolves in four days, with no recorded fatalities in clinical studies [25], likely due to pre-phase cytoreduction and vigilant monitoring. Daratumumab-related infusion reactions are generally mild and self-limiting, with no reported treatment-related deaths [18]. Data on outcomes of Carfilzomib-induced CRS are extremely scarce, with our case being the first reported instance and the only documented fatal outcome to date. Despite prompt recognition and receiving maximal supportive therapy with high dose steroids and tocilizumab the degree of cytokine activation was profound and precluded survival. While most patients tolerate Carfilzomib well, this case highlights the possibility of development of severe multiorgan failure that can mimic high-grade CAR-T toxicity, requiring intensive care and carrying a substantial mortality risk. Early recognition and aggressive management, including consideration of earlier tocilizumab or IL-1 antagonists, may be crucial in improving survival. Given the rarity and severity of this complication, further case reports are essential to better define risk factors and optimize treatment strategies to prevent fatal outcomes.

## 4. Conclusions

Given the consistent temporal association to dosing and by ruling out other possible causes we hypothesize that our patient died from, grade 5 cytokine release syndrome following Carfilzomib administration. This report highlights that even non-immunotherapy agents can trigger severe inflammatory responses and be fatal. The rapid progression to shock, ARDS, and multiorgan failure despite aggressive critical care and immunosuppressive therapy underscores the possibility of Carfilzomib-associated CRS. Given its clinical overlap with sepsis, and other infectious and inflammatory conditions, and the lack of specific test to diagnose it, keeping in mind broad differential diagnosis and rapidly initiating broad differential diagnostic work up is crucial. Every patient should receive empiric broad spectrum antimicrobial therapy until sepsis syndrome is ruled out. As Carfilzomib remains widely used in multiple myeloma treatment, further research and case reporting are needed to define risk factors, optimal management strategies, and preventive measures for CRS in this context.

## Figures and Tables

**Table 1:** Timeline of Carfilzomib Administration, Clinical Events, and Outcome. CBC—complete blood count; Hgb—hemoglobin; WBC—white blood cell count; Cr—creatinine; GFR—glomerular filtration rate; AST—aspartate aminotransferase; ALT—alanine aminotransferase; ICU—intensive care unit; MOF—multiple organ failure; IV—intravenous.

Timeline	Dose	Symptoms	Laboratory Findings	Treatment	Outcome
Day 0—First Dose of Carfilzomib	Carfilzomib 40 mg IV (20 mg/m^2^)	Fever, headache (CRS Grade 1)	**CBC**: Hgb 11.0, WBC 6.5, Lymphocyte count 0.99, Platelet count 106 **Renal**: Cr 1.4, GFR 54 **Liver**: AST 29, ALT 13	Acetaminophen	Symptoms resolved spontaneously
Day 7—Second Dose of Carfilzomib	Carfilzomib 110 mg IV (55 mg/m^2^)	Fever, mild hypotension, confusion (CRS Grade 2)	**CBC**: Hgb 9.2, WBC 5.0, Lymphocyte count 0.22, Platelet count 113 **Renal**: Cr 1.29, GFR 58 **Liver**: AST 21, ALT 18	1 L IV saline, acetaminophen, methylprednisolone	Mental status improved
Day 14—Third Dose of Carfilzomib	Carfilzomib 110 mg IV (55 mg/m^2^) + 125 mg IV methylprednisolone + 25 mg IV diphenhydramine	Refractory shock, multiorgan failure	**CBC**: Hgb 8.8, WBC 4.3, Lymphocyte count 0.19, Platelet count 82 **Renal**: Cr 1.21, GFR 63 **Liver**: AST 17, ALT 15	ICU admission, vasopressors, steroids, antibiotics	Rapid clinical decline
Day 14—6 h post-dose	-	Hypotension, tachycardia	**CBC**: Hgb 8.8, WBC 4.3, Platelet count 81	Vasopressors, empiric antibiotics	No clinical improvement
Day 14—12 h post-dose	-	Rising lactate, encephalopathy	**CBC**: Hgb 9.5, WBC 12.1, Platelet count 84	Hydrocortisone, tocilizumab	Progressive decline
Day 14—18 h post-dose	-	Refractory shock, ventilation initiated	**CBC**: Hgb 9.7, WBC 12.8, Platelet count 42	Mechanical ventilation	No response
Day 15	-	Cardiac arrest	-	Resuscitation unsuccessful	Death due to MOF following CRS

**Table 2:** Panel of Laboratory Examinations and Results on Blood, Bronchoalveolar lavage and CSF Samples obtained to evaluate various infectious, inflammatory, and autoimmune etiologies in febrile patient with bicytopenia and multiorgan failure with hyperacute hemodynamic decompensation following administration of carfilzomib. L—value under the lower threshold of the reference range; H—value above the upper threshold of the reference range.

**Cytokine Panel from the Blood**			
**Component**	**Reference Range**		**Value on Admission**
**TNF-alpha**	<10.0 pg/mL		28.9
**IL-6**	<5.0 pg/mL		651
**IL-10**	<7.0 pg/mL		13
**IFN-gamma**	<60.0 pg/mL		158
**GM-CSF**	<15.0 pg/mL		35
**CSF Pathogen Test Results**			
**Pathogen/Test**		**Result**	
**Gram stain**		Negative	
**Cultures**		Negative	
**Herpes simplex virus I and II PCR**		Negative	
**Varicella Zoster Virus PCR**		Negative	
**Cytomegalovirus PCR**		Negative	
**Human parechovirus PCR**		Negative	
**Human Herpesvirus 6 PCR**		Negative	
**Epstein Barr Virus PCR**		Negative	
**Enterovirus 71 PCR**		Negative	
**Adenovirus PCR**		Negative	
**LCM virus IgM and IgG**		Negative	
**West Nile Virus IgM and IgG Ab**		Negative	
**Jamestown Canyon Virus IgM Ab**		Negative	
**St. Louis Encephalitis Virus IgM and IgG Ab**		Negative	
**California (LaCrosse) Encephalitis Virus IgM and IgG**		Negative	
**West Equine Encephalitis Virus IgM and IgG Ab**		Negative	
**Human Immunodeficiency Virus PCR**		Negative	
**East Equine Encephalitis Virus IgM and IgG Ab**		Negative	
**Powassan Virus IgM, IgG, PRNT**		Negative	
**Escherichia coli K1 PCR**		Negative	
**Neisseria meningitidis PCR**		Negative	
**Borrelia burgdorferi PCR, IgM, IgG Ab**		Negative	
**Leptospirosis spp. IgM and IgG**		Negative	
**IGRA**		Negative	
**Listeria monocytogenes PCR**		Negative	
**Ehrlichia eauclairensis PCR**		Negative	
**Ehrlichia chaffensis IgM and IgG**		Negative	
**Anaplasma phagocytophilum IgG**		Negative	
**Anaplasma phagocytophilum IgM**		Negative	
**Anaplasma phagocytophilum PCR**		Negative	
**Borrelia miyamotoi PCR**		Negative	
**Streptococcus pneumoniae PCR**		Negative	
**Streptococcus agalactiae PCR**		Negative	
**Haemophilus influenza PCR**		Negative	
**VDRL**		Negative	
**Babesia microti PCR, IgG**		Negative	
**Cryptococcus neoformans/gattii PCR**		Negative	
**Tests Done from Sputum Obtained Through Bronchoalveolar Lavage**			
**Pathogen/Test**		**Result**	
**Pneumocystis jiroveci PCR**		Negative	
**Adenovirus**		Negative	
**Coronavirus 229E**		Negative	
**Coronavirus HKU1**		Negative	
**Coronavirus NL63**		Negative	
**Coronavirus OC43**		Negative	
**SARS Coronavirus-2**		Negative	
**Human Metapneumovirus**		Negative	
**Human Rhinovirus/Enterovirus**		Negative	
**Influenza A**		Negative	
**Influenza B**		Negative	
**Parainfluenza Virus 1**		Negative	
**Parainfluenza Virus 2**		Negative	
**Parainfluenza Virus 3**		Negative	
**Parainfluenza Virus 4**		Negative	
**Respiratory Syncytial Virus**		Negative	
**Bordetella parapertussis**		Negative	
**Bordetella pertussis**		Negative	
**Chlamydia pneumoniae**		Negative	
**Mycoplasma pneumoniae**		Negative	
**Blood Tests**			
**Pathogen/Test**		**Results**	
**Babesia microti PCR**		Negative	
**Babesia duncani PCR**		Negative	
**Babesia divergens/MO-1 PCR**		Negative	
**Borrelia burgdorferi PCR**		Negative	
**Anaplasma phagocytophilum PCR**		Negative	
**Ehrlichia chaffensis PCR**		Negative	
**Borrelia miyamotoi PCR**		Negative	
**Human Immunodeficiency Virus PCR**		Negative	
**Epstein Barr Virus PCR**		Negative	
**Cytomegalovirus PCR**		Negative	
**Hepatitis A, B, C serology**		Negative	
**Leptospira spp. serology**		Negative	
**Blood culture**		Negative	
**RPR**		Negative	
**Ferritin**		850 ng/mL	
**Triglyceride**		120 mg/dL	
**Fibrinogen**		210 mg/dL	
**CD-25 (soluble IL-2 receptor)**		1800 U/mL	
**Urine Tests**			
**Test**		**Result**	
**Urine culture**		Negative	

**Table 3:** Most important differential diagnosis consideration in a febrile patient with pancytopenia and evidence of SIRS. HLH—hemophagocytic lymphohistiocytosis; IRIS—immune reconstitution inflammatory syndrome; TAFRO—thrombocytopenia, anasarca, fever, reticulin fibrosis, organomegaly; TLS—tumor lysis syndrome.

Diagnosis	Etiology	Timing	Specific Work-Up	Cytokine Panel	Treatment
**CRS**	- CAR T cell therapy - Immune checkpoint inhibitor therapy	Up to a few weeks post infusion	- IL-6 - CRP - Ferritin	- IL-6 ↑↑ - IFN-γ ↑ - TNF-α ↑	- Tocilizumab - Corticosteroids - Supportive care (fluids, oxygen, vasopressors if needed) - Discontinue/pause immunotherapy
**HLH**	- Primary - Secondary: (1) Infection (2) Malignancy (3) Autoimmune diseases	Most common in early childhood, but anytime	- Ferritin - Soluble IL-2 receptor (sCD25) - NK cell activity - Bone marrow biopsy	- IL-6 ↑ - IFN-γ ↑↑ - IL-2R ↑↑	- Dexamethasone - Etoposide - Cyclosporine A - Anakinra/emapalumab - Treatment of underlying cause - Hematopoietic stem cell transplant
**Sepsis**	- Mostly bacterial infections - Rarely viral and fungal infections	Rapid onset within hours in critically ill and immunocompromised, fast onset in vulnerable populations (e.g., elderly, neonates). Depends on the source of infection	- Blood cultures x 2 - Lactate - CRP, procalcitonin - Urinalysis + urine culture	- IL-6 ↑ - TNF-α ↑	- Broad spectrum antibiotics - IV fluids - Vasopressors - Source control - Supportive care
**IRIS**	- AIDS with immune reconstitution	After CD4+ cell count increase following therapy	- CD4 count and HIV viral load - Cultures or PCRs for known pathogens (TB, MAC, CMV, cryptococcus, etc.) - Tissue biopsy—if localized lesion is present	- IL-6 ↑ - IFN-γ ↑ - IL-2R ↑	- Continue ART - Corticosteroids - Treatment of underlying infections - Supportive care
**TAFRO**	- Idiopathic - Immune dysregulation	Rapidly progressive discourse, evolving over days to 1–3 weeks	- IL-6 - Bone marrow biopsy - CT imaging	- IL-6 ↑↑	- Corticosteroids - Tocilizumab - Rituximab - Cyclosporine A - Supportive care
**TLS**	- Hematologic malignancies with high tumor burdens and rapid proliferation - Cytotoxic chemotherapy or radiation therapy	Very rapid onset within 12–72 h after initiating cytotoxic therapy	- Serum uric acid - Serum phosphate - Potassium	- IL-6 ↑	- Aggressive IV hydration - Allopurinol - Rasburicase - Electrolyte correction - Dialysis
**SARS-CoV-2 Cytokine Storm**	- Severe SARS-CoV-2 infection	Typically within the second week of illness (around day 7–10)	- IL-6 - Ferritin - CRP	- IL-6 ↑↑ - IL-2R ↑	- Corticosteroids - Tocilizumab - Antivirals - Anticoagulation therapy - Supportive care

## Data Availability

The data that support the findings of this study are available from the corresponding author upon reasonable request.
